# Behçet’s disease and tuberculosis: unmasking infection behind a suspected flare

**DOI:** 10.1590/S1678-9946202567052

**Published:** 2025-08-18

**Authors:** Jobson Lopes de Oliveira, Igor Albuquerque Nogueira, Maurício Catunda Pinheiro Jucá, Afonso Rocha Eisele, Luís Arthur Brasil Gadelha Farias, Diana Arrais de Souza Rangel

**Affiliations:** 1 Centro Universitário Christus, Faculdade de Medicina, Fortaleza, Ceará, Brazil; 2 Universidade Federal do Ceará, Faculdade de Medicina, Programa de Pós-Graduação em Ciências Médicas, Fortaleza, Ceará, Brazil; 3 Universidade de São Paulo, Faculdade de Medicina de Ribeirão Preto, Hospital das Clínicas, Ribeirão Preto, São Paulo, Brazil; 4 Hospital São José de Doenças Infecciosas, Fortaleza, Ceará, Brazil; 5 Universidade de São Paulo, Faculdade de Medicina, Hospital das Clínicas, Departamento de Doenças Infecciosas, Laboratório de Investigação Médica (LIM-49), São Paulo, São Paulo, Brazil; 6 Hospital de Messejana Dr. Carlos Alberto Studart Gomes, Divisão de Pneumologia, Fortaleza, Ceará, Brazil.

**Keywords:** Behçet’s disease, Tuberculosis, Mycobacterium tuberculosis, infection, Lymphadenopathy

## Abstract

Behçet’s disease (BD) is a chronic and multisystem disorder characterized by recurrent oral and genital ulcers, along with ocular, cutaneous, vascular, gastrointestinal, and neurologic manifestations. The etiology is thought to involve an autoimmune response triggered by infectious or environmental factors in genetically predisposed individuals. *Mycobacterium tuberculosis* has been proposed as a potential trigger for BD, although this association remains rarely reported. We show a compelling case of a patient with BD diagnostic criteria who subsequently developed mediastinal tuberculous lymphadenitis, which was initially suspected as disease activity. This case underscores the importance of considering tuberculosis in BD patients with new or worsening symptoms despite appropriate therapy.

## INTRODUCTION

Behçet’s disease (BD) is a chronic autoinflammatory disorder that affects multiple organ systems, including the eyes, oral and genital mucosa, skin, and gastrointestinal and neurological systems, with a relapsing-remitting course^1^. This is most prevalent in regions along the historic Silk Road, including the Mediterranean, Middle East, and Far East. The disease typically manifests in the third decade of life and males often experience more severe symptoms^2,3^.

Diagnosis is based on a clinical criteria established by the International Study Group (ISG), which require recurrent oral ulcers and at least two of the following manifestations as mandatory features: genital ulcers, ocular lesions, cutaneous lesions, or a positive pathergy test^4^. The more recent International Criteria for Behçet’s Disease (ICBD) include neurological and vascular involvement, providing greater sensitivity (Table 1)^5^.

**Table 1:** ISG (1990) and ICBD (2014) criteria for diagnosis of Behçet’s disease.

CRITERIA	ISG criteria^4^	ICBD criteria^5^	POINTS
	CONSIDERATIONS	SIGN/SYMPTOM	
**Recurrent oral ulceration**	Minor aphthous, major aphthous, or herpetiform ulcers observed by the physician or patient, which have recurred at least three times over a 12-month period	**Ocular lesions Genital aphthosis Oral aphthosis Skin lesions Neurological manifestations Vascular manifestations Positive pathergy test**	2 2 2 1 1 1 1
**Plus any two of the following:**		Scoring ≥ 4 indicates Behçet’s diagnosis	
**Recurrent genital ulceration**	Aphthous ulceration or scarring observed by the physician or patient		
**Eye lesions**	Anterior uveitis, posterior uveitis, or cells in the vitreous on slit lamp examination; or retinal vasculitis detected by an ophthalmologist		
**Skin lesions**	Erythema nodosum observed by the physician or patient, pseudofolliculitis, or papulopustular lesions; or acneiform nodules observed by the physician in a post adolescent patient who is not receiving corticosteroids		
**Positive pathergy test**	An indurated erythematous small papule or pustule after intradermal puncture of the skin with a 20-gauge needle 5 mm obliquely into the patient’s flexor aspect of the avascular forearm skin under sterile conditions and without injecting saline. Test interpreted as positive by the physician at 24-48 h.		

The pathogenesis of BD remains unclear but likely involves genetic predisposition (e.g., HLA-B51), environmental triggers, and immune dysregulation. Infectious agents such as *Herpes simplex virus-1*, *Streptococcus sanguinis*, and *Mycobacterium tuberculosis* have been implicated, although their exact role is uncertain^1,6,7^.

Tuberculosis (TB) remains a global health challenge, which predominantly affects the lungs but is also associated with autoimmune-like phenomena following pathogen exposure. The potential role of TB in triggering BD and the contribution of immune dysregulation to its pathogenesis remain poorly understood. The complex relationship between these two diseases, particularly concerning causality and shared pathological pathways, needs further investigation to clarify underlying mechanisms and clinical implications^7,8^.

This report describes a case of BD complications due to mediastinal tuberculous lymphadenitis, with an analysis of the diagnostic and pathophysiological relationship between these coexisting conditions. The patient provided written informed consent for participation in the study and publication of anonymized case details, including accompanying images.

## CASE REPORT

**Figure 1:**
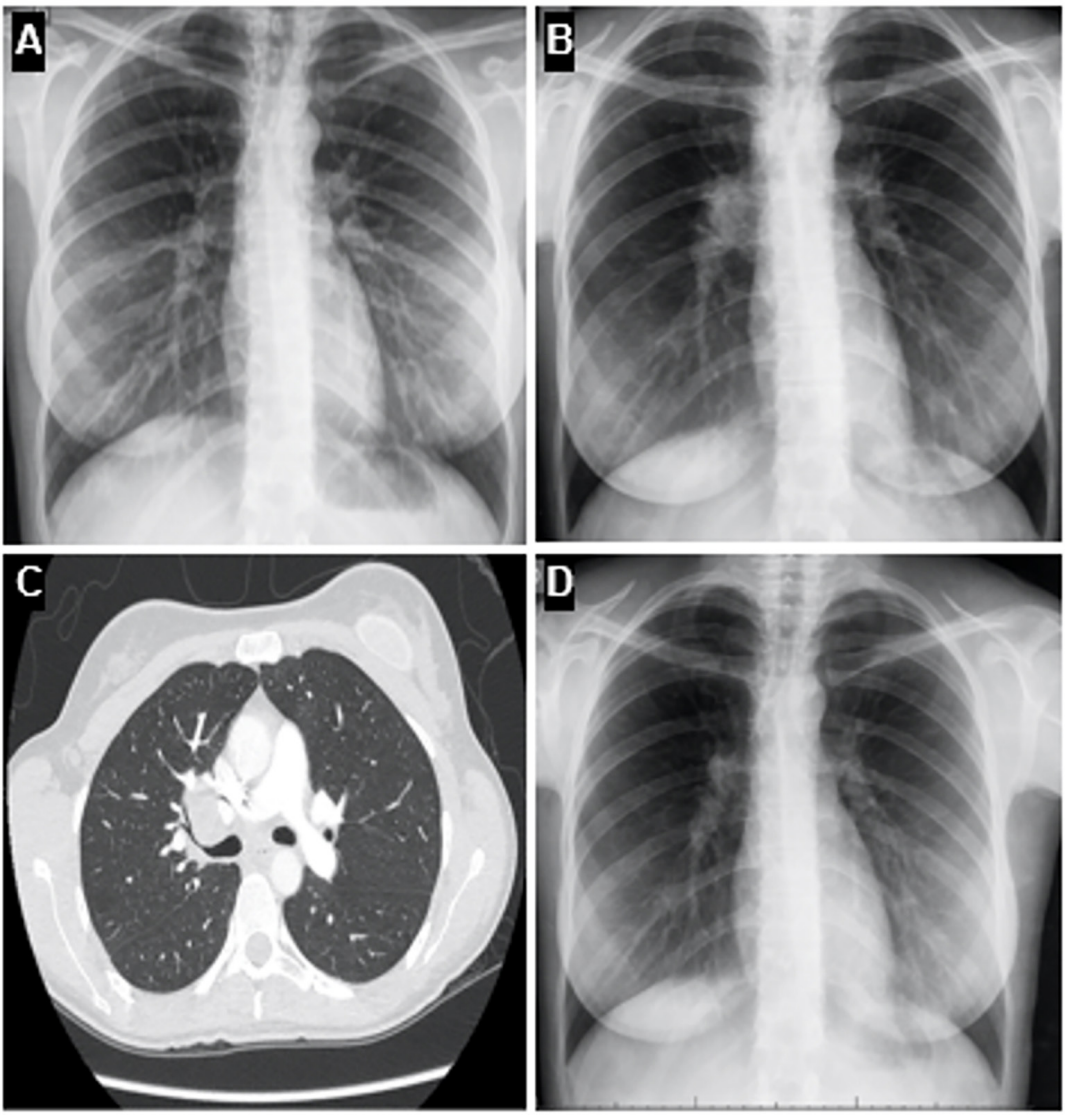
Serial chest imaging: (A) Upon initial diagnosis of Behçet’s disease; (B) During recurrence of oral ulcers, with concurrent fever and weight loss, showing right hilar enlargement; (C) Axial chest CT demonstrates enlarged paratracheal and subcarinal lymph nodes compressing the right main bronchus; (D) Post-completion of anti-tuberculosis therapy.

A 27-year-old White female physician from Brazil had a 12-month history of recurrent oral and genital ulcers, pseudofolliculitis, and erythema nodosum-like lesions. Her medical history included atopic dermatitis and well-controlled asthma, and she did not have personal or family history of TB but had received childhood BCG vaccination. The initial evaluation revealed elevated inflammatory markers, whereas serology for syphilis, HIV, cytomegalovirus, and HSV-1/2 were negative. Then, a chest radiograph was unremarkable (Figure 1A).

BD was diagnosed based on ISG and ICDG criteria and the ophthalmologic examination ruled out ocular involvement. The treatment with colchicine and prednisone (the latter was initiated at 40 mg/day and followed by a 6-month tapering regimen) achieved complete resolution of the cutaneous lesions. Neither tuberculin skin test (TST) nor interferon-gamma release assay (IGRA) screening for latent tuberculosis infection (LTBI) was performed prior to the immunosuppressive therapy.

**Figure 2:**
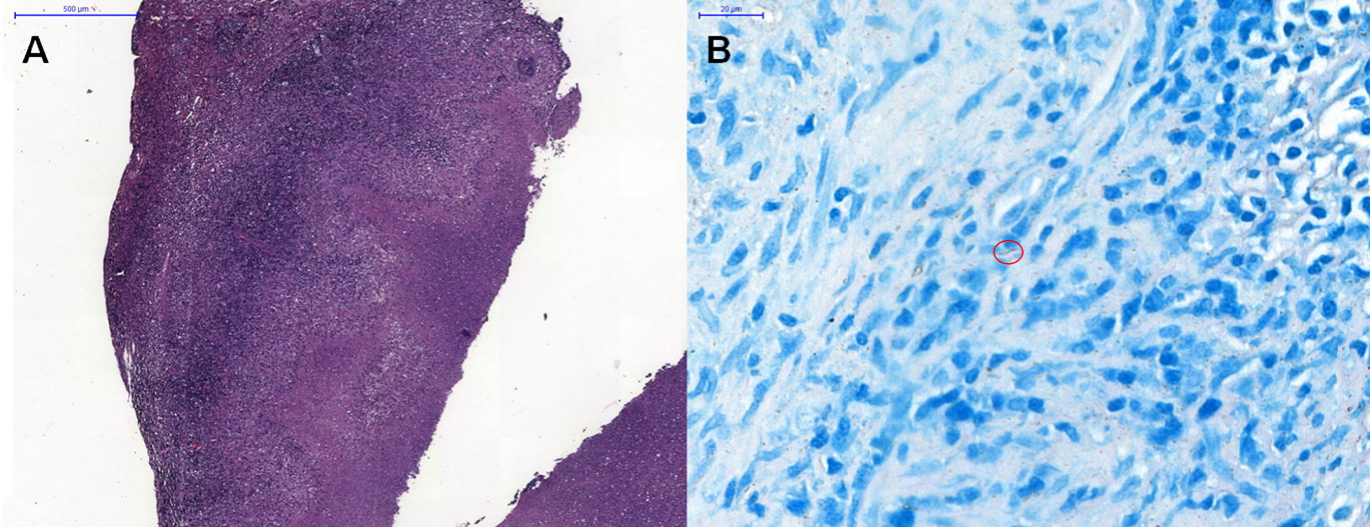
(A) Lymph node histopathology demonstrating caseous granuloma with central necrosis and surrounding lymphohistiocytic infiltrate (50× magnification); (B) Acid-fast bacilli (red circle) identified via Ziehl-Neelsen staining (650× magnification).

Two years later, the patient developed recurrent oral ulcers (despite continued use of colchicine and topical steroids), erythema nodosum-like lesions on the chin, low-grade fever, dry cough, and had a weight loss of 4 kg. The chest radiography revealed right hilar enlargement (Figure 1B), and the computerized tomography (CT) of the chest showed mediastinal and hilar lymphadenopathy compressing the right main bronchus (Figure 1C). Lymph node biopsy showed granulomatous inflammation with caseation and no malignancy (Figure 2A). Ziehl-Neelsen staining was positive for acid-fast bacilli (Figure 2B), and cultures confirmed *M. tuberculosis*, which established the diagnosis of mediastinal tuberculous lymphadenitis.

The patient received standard anti-TB therapy (rifampicin, isoniazid, pyrazinamide, and ethambutol for two months, followed by rifampicin and isoniazid for four months), with significant clinical and radiological improvement (Figure 1D). She remains in BD remission on colchicine 2 mg/day, without TB recurrence.

## DISCUSSION

This case underscores the complexity of the diagnostic of new-onset symptoms in BD, particularly hilar enlargement, which needs prompt evaluation for pulmonary artery aneurysm (PAA), a severe vascular manifestation, alongside infectious or neoplastic etiologies. PAA, which is observed in 1%-33% of BD cases (predominantly young males), requires urgent differentiation due to their life-threatening potential. Contrast-enhanced CT serves as the cornerstone for assessing vascular and parenchymal pulmonary involvement. Following exclusion of vascular pathology, lymph node biopsy becomes critical to differentiate TB from malignancy^9,10^. In this patient, TB was confirmed histologically and microbiologically.

A growing body of evidence has shed light on mechanisms underlying the association between *M. tuberculosis* infection and BD. First, molecular mimicry between *M. tuberculosis* heat shock protein (HSP) and human HSP may induce cross-reactive immune responses, potentially contributing to BD pathogenesis^11,12^. Additionally, impairments in cell-mediated immunity observed in BD patients could heighten susceptibility to TB^13,14^. Genetic factors also play a role: HLA-B51, a major genetic risk factor for BD, is linked to increased susceptibility to pulmonary TB, whereas HLA-B52 shows a protective association^15^. Finally, immunosuppressive therapies used in BD management may elevate the risk of TB^14^.

The co-occurrence of TB and BD poses diagnostic and therapeutic challenges. In a Chinese cohort, 5.4% of hospitalized BD patients had active TB, which correlated with increased systemic symptoms^16^. LTBI is also prevalent in BD; a retrospective study of 232 BD patients identified LTBI in 29.3%, in which the BD-LTBI subgroup showed more severe oral/genital ulcers and ocular involvement compared to non-LTBI counterparts^17^. IGRA may aid TB diagnosis in BD, as elevated spot-forming cell counts correlate with active infection^18^.

Notably, microbial infections, including *M. tuberculosis*, can trigger a BD-like syndrome characterized by overlapping clinical features (e.g., oral/genital ulcers, arthritis), which is typically solved with antimicrobial therapy rather than immunosuppressants^13^. A review by Zhang et al. highlights that such cases predominantly affect females (mean age: 38.6 years), with genital ulcers (100%), oral ulcers (86%), and arthritis (71%) as common manifestations, but lack major organ involvement^19^. Conversely, in confirmed BD patients, concurrent TB may exacerbate disease activity, and anti-TB therapy alone often fails to induce remission, requiring combined immunomodulatory strategies^13^.

A great gap in the pre-therapeutic evaluation highlighted by this case was the absence of LTBI screening prior to initiating immunosuppressive therapy. According to the Brazilian Recommendations Manual for Tuberculosis Control, screening for LTBI using either TST or IGRA is recommended for individuals starting tumor necrosis factor (TNF) inhibitors or corticosteroids at doses exceeding 15 mg/day of prednisone (or equivalent) for more than 30 days^20^. In this case, the high initial dose and extended duration of corticosteroid therapy clearly met the criteria mandating LTBI screening and, if indicated, preventive treatment. The omission of this evaluation constitutes a critical lapse in care, underscoring the imperative for systematic LTBI screening before immunosuppressive therapy, particularly in high-burden settings. This oversight might have contributed to the subsequent development of active tuberculosis in this patient.

## CONCLUSION

Proactive TB screening is essential in BD management, particularly in high-prevalence regions, and should be performed before initiating corticosteroids or immunosuppressive therapies. The initial screening should include TST or IGRA for LTBI detection, complemented by chest radiography. Advanced imaging is crucial to differentiate TB from BD-related complications (e.g., PAA) when there are pulmonary manifestations. Finally, in refractory cases, physicians should maintain a high index of suspicion for occult infections before attributing symptoms to BD activity.
